# Articles of Scientific Faith

**Published:** 1891-06-27

**Authors:** 


### The Hospital

A Weekly Journal of

Science, Medicine, Nursing, and Philanthropy.

*For Hospitals, Asylums, Infirmaries, Dispensaries, Medical Practitioners, Students, Nurses, and the Charitable Public.*

Vol. X.—No. 248.] [June 27, 1891.

### Articles of Scientific Faith.

Certain recent events have transpired which, though they are of no great public importance in themselves, and, therefore, need not be particularly mentioned, are yet worth considering because they show that even among educated persons the common-places of scientific method are comparatively little understood and much less practised. Now the true scientist is, perhaps, the sternest intellectual worker of the present time, and he is also in more deadly earnest than almost any other educated man. It is the strenuous and earnest men who most powerfully move and influence their contemporaries. No matter how much truth a man may have to tell, or how great in itself may be the work which he has on hand, if he be a person of indifferent temper and languid activity, neither his truth nor his work will be of much significance to men who are busily engaged in other affairs. The true scientist is not a man of indifferent temper or languid activity. He is, as we have said, tremendously in earnest. He is impressed with the conviction that he lives in a foolish and an ignorant world: that the bulk of his fellow-men have very little knowledge of many things which they ought to know well and thoroughly, and that the little knowledge they do actually possess is so crude and unintelligent that it is almost worse than no knowledge at all.

There is a ring of contempt in the tone of the scientist when he speaks of the unscientific crowd. Mr. Herbert Spencer, it is said, has long ago given up the idea that the brains of the average man, even of the average man of culture, are of any great account. We know by repeated experience what a magnificent contempt Professor Huxley feels for the scientific and reasoning capacity of the Duke of Argyll. It is equally clear that Mr. John Tyndall regards certain exponents of theological orthodoxy as little better than that familiar denizen of the village common whose function it is to bray.

None of us would say any other than that Mr. Herbert Spencer, Professor Huxley, and Mr. John Tyndall are able and weighty men. They cannot be regarded with indifference, still less with contempt. It is interesting, and it is important too, to consider how such really able and weighty men as these come to have a kind of anger and scorn for their educated contemporaries. Commonplace mortals may readily dispose of the question by attributing the scorn of scientific men to their vanity and to their deficiency in knowledges other than their own. But that would not be either a true or a sufficient explanation.

What is the real reason why distinguished scientists, unless they be men of peculiarly magnanimous temper, like Faraday, are so impatient and contemptuous of the opinions of their unscientific contemporaries? This is the reason: They know that they themselves pay the full price for the truth which they become possessed of, whilst the unscientific either do not care to possess themselves of scientific truth at all, or, if they do, always try to acquire it by the process of appropriation known as stealing. In other words, the true scientist gains his wealth by open, recognised, and honest scientific methods; and having so gained it, he knows the full and exact value of it. The unscientific gains his truth as the old-fashioned highwayman acquired his wealth, by riding forth on a more or less trusty steed and picking up incidentally whatever he can find on the expedition.

One of the prime articles of the faith of the scientist is "first-hand knowledge." Hearsay, hypothesis, the *a priori* opinions of men with a gift of tongues—these are his abominations. But when a man takes the trouble not only to eat a slice of bread, but also to go to the baker's and see it made and baked, to the miller's to see the corn ground and milled, to the farmer's to see the seed sown and the crop reaped and threshed, to the botanist's in order to learn the nature and affinities of the corn plant, to the chemist's laboratory to find out not only how the grain of corn is built up and organised, but what may be the chemical character of every element of which the grain consists—when a man does all these things, or most of them, for himself, it is clear that he possesses a knowledge of bread and corn, and has earned a right to speak on the subject, which does not appertain to him who only knows that the baker supplies the loaf at so much a pound and that the article is suitable for making nursery puddings and buttered toast. The scientist believes in himself and in the knowledge he possesses because he has worked, so to speak, with his own hands, and certainly with his own brain, and eyes, and nose, and other senses, and has found out for himself what is exactly the nature of the thing, in all its parts, which he ventures to talk about. The scientist works among things first of all. To him books and hearsay may be very good candles, and even electric lights, for helping him at his work, but they are not the work itself. The things and the things alone, and the knowledge he has gained by handling them, constitute the truth he has to deal with. When, therefore, some other man presents himself, who knows nothing of actual things, very little of books, and has but a mere smattering even of newspaper articles and current talk, how can it be otherwise but that the true scientist will regard his pretences to knowledge with anger and contempt?

The scientist regards as one of the fundamental articles of his creed what he calls a "sufficient induction." It is surprising what a number of educated men, and even of clever men, there are who seem to regard the method of Bacon as entirely superfluous. The writer has in his mind at the present moment a newspaper editor, a senior officer of Her Majesty's army, and a clever and original surgeon, each of whom he has known to make a single trial of a certain pill or mixture, and thenceforward to swear by it as practically infallible for its particular purpose. Now to a real scientist this is simply silly; it is the very essence of inconsequence. What the scientist does is not to make a single trial of a thing, but repeated trials; not one nor half-a-dozen only, but fifty or five hundred, or ten thousand if possible. Not only does he make numerous trials, but he takes care that the trials are always made under similar circumstances, and that every possible source of fallacy, whether great or small, is entirely taken away. When a man pursues his work in this fashion, he knows what he is doing, and what he is talking about. The flippant guesser, the self-confident theory-monger he scorns, just as the experienced horseman scorns the Cockney apprentice who takes a penny ride on the back of a donkey at Hampstead on a Bank Holiday.

It might be supposed that there is no particular novelty in sticking to the bare truth. But the scientist finds it absolutely necessary to make it an article of his faith that the observer who pretends to science shall always report exactly what he sees and nothing else. The very foundation of science is fact, and nothing but fact; things exactly as they are, or, at least, as they appear to the trained observer. The scientist, of course, is not a mere reporter of facts, he is a reasoner as well. But he makes the sharpest possible distinction between his statement of fact and the theories he may found thereupon. He may be the most prejudiced reasoner in the world without losing his character as a scientific man; but the moment he begins to misstate facts he is scientifically ruined; he sinks himself at once to the base level of a perjured witness. Science hates and despises him, and casts him out of her communion with all speed. Among the Jews, when certain offences were committed, Moses commanded that the offender should be stoned. Science does not stone her lying apostates, but she would if she could; and this extreme degree of anger is entirely right and just.

Some readers may be surprised to find that in the articles of the faith of the scientist as here set forth, the want of novelty is exceedingly conspicuous. According to this view, the chief factors in the scientist's methods of procedure are industry, intelligence, and honesty. That is so. The scientist is doing his best to regenerate his age by these plain and simple principles. The theologian, who speaks so much of another kind of regeneration, would do well to think occasionally of the new birth of the scientist—the birth into stern work, unprejudiced intelligence, and unflinching honesty. There is a great deal in this kind of regeneration. It is a gospel that is emphatically worthy to be preached; and it demands for its propagation preachers much above the level of the average theologian. The scientist's faith, in short, rightly understood, has in it the promise of a new life for the world.

